# Prevalence of high risk of ADHD among adult users of Instagram in Russian Federation

**DOI:** 10.1192/j.eurpsy.2022.1461

**Published:** 2022-09-01

**Authors:** S. Potanin, M. Morozova, G. Rupchev, D. Burminskiy, T. Lepilkina, A. Beniashvili

**Affiliations:** 1 FSBI Research Center of Mental Health, Laboratory Of Psychopharmacology, Moscow, Russian Federation; 2 Moscow State University, Faculty Of Psychology, Moscow, Russian Federation

**Keywords:** Adult ADHD Self-Report Scale, adult population, Instagram, adhd

## Abstract

**Introduction:**

The problem of diagnosis and treatment of ADHD in adults in the Russian Federation remains very relevant. Unfortunately, very little time is devoted to the topic of adult ADHD in standard and advanced training programs. In connection with these problems, it is important to study the prevalence of ADHD in the Russian population.

**Objectives:**

To determine the prevalence of an increased risk of adult ADHD on the example of Russian Instagram users.

**Methods:**

In the professional blog of one of the authors of the article in Instagram (@gentlepsydoc), a link was posted to an anonymous survey conducted using Google docs, in which patients filled out the Adult ADHD Self-Report Scale (ASRS v1.1) in the author’s translation

**Results:**

A total of 144 people took part in the screening, 87.5% of them were women, the average age of the participants was 35.5±12.3 years (from 20 to 56). An increased risk of ADHD according to the indicators of the ASRS scale was detected in 13.2 % of participants.

**Conclusions:**

Among Instagram users, the prevalence of an increased risk of ADHD was higher than according to epidemiology data. This indicator could be influenced by a sample of patients, mainly consisting of subscribers of a professional blog of a psychiatrist, as well as the prevalence of women among the participants. In any case, these data speak in favor of the importance of diagnosing this condition in the Russian population, as well as including information about adult ADHD in training programs for mental health professionals.

**Disclosure:**

No significant relationships.

